# LV function validation of computer-assisted interventional system for cardiac resyncronisation therapy

**DOI:** 10.1007/s11548-018-1748-0

**Published:** 2018-03-30

**Authors:** Maria Panayiotou, R. James Housden, Athanasius Ishak, Alexander Brost, Christopher A. Rinaldi, Benjamin Sieniewicz, Jonathan M. Behar, Tanja Kurzendorfer, Kawal S. Rhode

**Affiliations:** 1 Division of Imaging Sciences and Biomedical Engineering, King’s College London, London, UK; 2 Siemens Healthineers, Forchheim, Germany; 3 Department of Cardiology, Guy’s and St. Thomas’ Hospitals NHS Foundation Trust, London, UK

**Keywords:** Cardiac resynchronisation therapy, Left ventricular volumes, Ejection fraction, Cardiac magnetic resonance imaging

## Abstract

**Purpose:**

Cardiac resynchronisation therapy (CRT) is an established treatment for symptomatic patients with heart failure, a prolonged QRS duration, and impaired left ventricular (LV) function; however, non-response rates remain high. Recently proposed computer-assisted interventional platforms for CRT provide new routes to improving outcomes. Interventional systems must process information in an accurate, fast and highly automated way that is easy for the interventional cardiologists to use. In this paper, an interventional CRT platform is validated against two offline diagnostic tools to demonstrate that accurate information processing is possible in the time critical interventional setting.

**Methods:**

The study consisted of 3 healthy volunteers and 16 patients with heart failure and conventional criteria for CRT. Data analysis included the calculation of end-diastolic volume, end-systolic volume, stroke volume and ejection fraction; computation of global volume over the cardiac cycle as well as time to maximal contraction expressed as a percentage of the total cardiac cycle.

**Results:**

The results showed excellent correlation ($R^{2}$ values of $>\,0.99$ and Pearson correlation coefficient of $>\,0.98$) with comparable offline diagnostic tools.

**Conclusion:**

Results confirm that our interventional system has good accuracy in everyday clinical practice and can be of clinical utility in identification of CRT responders and LV function assessment.

## Introduction

Cardiovascular disease is the main cause of morbidity and mortality worldwide with a lifetime risk of heart failure of one in five [28]. Cardiac resynchronisation therapy (CRT) has been established as an effective treatment for patients with symptomatic chronic heart failure associated with left ventricular (LV) dyssynchrony [1]. Despite advances in medical devices, approximately 30% of patients are inadequate responders to CRT [32]. One promising new avenue for improving CRT is the recent development of computer-assisted interventional platforms [3, 25]. Such platforms extract clinically meaningful parameters and planning information from pre-operative data and use this information for image-guided interventions.

LV function is one of the principal parameters of interest in planning and guiding CRT procedures. It can be derived from imaging modalities such as US or MRI. However, not all imaging modalities are appropriate for planning/guidance and diagnostic tools are often unsuitable for computer-assisted interventions which require fast, highly automated and easy to use software.

Two-dimensional (2D) echocardiography for the assessment of ejection fraction (EF) [31] was the most widely used for many years because it offers fast, relatively inexpensive and non-invasive functional analysis. However, this technique has several limitations such as operator dependence, the dependence on geometric assumptions [9], the inadvertent use of foreshortened views, the restriction to only two planes and suboptimal endocardial border detection [17, 20].

In an attempt to overcome these severe limitations, real-time three-dimensional echocardiography (RT3DE) has been developed which shows promise for more accurate LV evaluation [10, 16, 24, 34], as it does not rely on geometric assumptions for volume calculations and is not subject to plane positioning errors which can lead to chamber foreshortening [21]. RT3DE also captures the entire volumes, which is of great importance in deformed ventricles [14].

Compared with cardiac magnetic resonance (CMR) imaging, LV volumes calculated from RT3DE showed significantly smaller bias and lower intra- and interobserver variability than 2D echocardiography [21]. However, there are inherent limitations with echocardiography such as variable image quality, time-consuming workflow and associated problems with reproducibility and these may impact the integrity of associated measures of ventricular function [26, 30, 32, 35]. Previous studies have demonstrated that LV volumes and EF measurements using RT3DE are accurate when compared with CMR imaging only in patients with optimal image quality [11, 13]. However, in patients with poor acoustic windows, relatively low correlations were noted despite the use of contrast enhancement [8]. An additional critical disadvantage of echocardiography when compared to CMR imaging is its inability to visualise myocardial scar. During CRT, placement of the LV lead away from myocardial scar is an important factor that contributes to CRT response.

Magnetic resonance imaging (MRI) is considered the method of choice and the reference standard for global and regional myocardial function assessment [4]. CMR provides high reproducibility, low variability, superior image quality and complete LV coverage [7], as well as information about the position and extent of myocardial scar [5]. Measures of dyssynchrony can be derived from cine and tagged images looking at volume change, myocardial thickening and strain [6, 12, 19, 22, 23]. Cardiac MRI-measured myocardial strain is highly reproducible and accurate [36–38]. However, strain assessment often requires dedicated imaging techniques with advanced processing [15, 18, 27, 38]. For that reason, such techniques are commonly applied in research settings but rarely used in routine clinical practice. Additionally, a study performed by Sohal et al. [32] on investigating measures of mechanical dyssynchrony as a predictor of CRT found that volume-change systolic dyssynchrony index has superior sensitivity and specificity compared to other mechanical dyssynchrony measures for predicting chronic reverse remodelling.

In a research setting, experimental or retrospective clinical studies can use diagnostic tools (e.g. TomTec or CVI42) to process pre-operative data offline. However, translating research into usable clinical systems requires dedicated computer-assisted interventional software. Interventional tools have fundamentally different requirements from diagnostic tools: they must be fast, highly automated and be easy to use for the interventional team who may not be trained to interpret pre-operative data. These demanding requirements are illustrated in a computer-assisted CRT intervention performed in a MAGNETOM Artis Combi Suite [3]. The pre-operative MRI is acquired immediately before the X-ray-guided intervention. This is an attractive workflow for the patient, clinical team and hospital. However, all planning and guidance information must be processed in the short time it takes to move the patient from the MRI to the adjacent cath lab.

The aim of this retrospective study is to evaluate a fast, highly automated computer-assisted interventional platform for CRT [3, 25] against offline diagnostic tools. This interventional CRT platform includes a planning stage which allows planning of the procedure to be performed within minutes before the X-ray-guided intervention. In CRT, patients are selected by functional criteria: normal sinus rhythm with EF $\le$ 35%, symptomatic heart failure (NYHA functional class III/IV) on maximal achievable medical therapy and QRS prolongation ($\ge$ 120 ms) with LBBB pattern on ECG. In the planning stage of our interventional approach, end-diastolic volume (EDV), end-systolic volume (ESV), stroke volume (SV) and hence ejection fraction (EF) are computed. These parameters, particularly EF, are essential predictors of CRT outcome and consequently could decrease the number of inadequate responders to CRT.

**Fig. 1:**
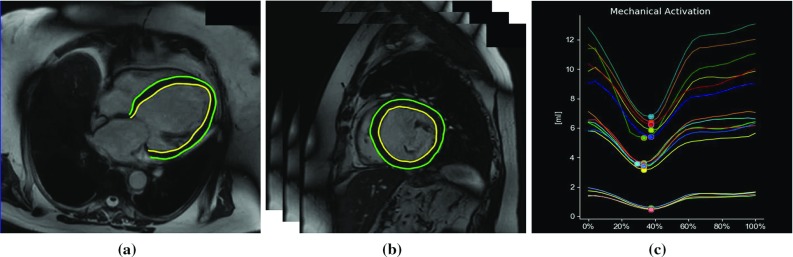
Pre-procedural **a** LA and **b** SA cine MRI acquisition, along with automatic outline of endocardial (yellow) and epicardial (green) contours in the acquired SA and LA images. **c** Display of regional volume curves expressed as a percentage of the total cardiac cycle for each of the 16 segments of the American Heart Association LV model

Our interventional approach is not limited to computing functional parameters. The novel system uses standard clinical MRI to quantify scar and compute regional volume analysis as well. This information assists doctors to identify target areas for the deployment of the LV lead. In addition, a guidance step is included, where the pre-operative planning data are overlaid on X-ray images and motion compensated to guide the procedure and improve the likelihood of a patient responding to the treatment. However, this paper only focuses on the planning stage of the procedure, and more specifically on showing that global myocardial volumes and LV function can be accurately computed in a time critical and challenging interventional workflow. A comparison is performed against two offline diagnostic tools: circle CVI 42 (CMR) and TomTec (RT3DE). The evaluation is performed on 16 patients and 3 healthy volunteers.

## Methods

We acquired data from 3 healthy volunteers and 16 patients with heart failure and conventional criteria for CRT. Eleven of those patients were processed with all approaches: the two software systems circle CVI 42 and TomTec and the our interventional system. The rest of the patients and the 3 volunteers were only processed with circle CVI42 and our interventional system. Patients underwent both RT3DE and CMR imaging within 2 weeks to minimise changes in cardiac function. No patients or segments were excluded because of poor image quality.

### Image acquisition protocol

*Cardiac magnetic resonance imaging* Patients were scanned using a 1.5T MR scanner (MAGNETOM Aera, syngo MR E11, SIEMENS Healthcare, Germany). A multiple-slice cine steady-state free precession (SSFP) scan was performed in a stack of short-axis (SA) slices covering the LV and in the 4-, 3- and 2-chamber orientations.

*Real-time three-dimensional echocardiography* RT3DE studies were performed using an ultrasound machine (LOGIQ E9 Ultrasound Machine, VE98833, GE Healthcare, USA). A full minimum standard transthoracic echocardiography was performed in the SA and 2- and 4-chamber long-axis (LA) orientations. Additionally, a 3D clip of the LV was performed to measure 3D EF and LV volumes.

### Image analysis

*CMR imaging: Computer-assisted interventional system* [3, 25] Following the pre-procedural CMR acquisition, a fully automatic slice-by-slice segmentation and propagation of the endocardial and epicardial LV borders, at each time point in the stack of SA and three LA SSFP cine images, was computed. The epicardial and endocardial surfaces of the LV are extracted automatically using a model-based segmentation algorithm [2]. Spatial contour re-positioning is used to correct for any misalignment of the segmented SSFP endocardial and epicardial borders (Fig. 1a, b). Any contours found in any slices where the mitral valve is visible are excluded from the segmentation. The LV segmentation step has been previously quantitatively evaluated on 14 CRT patients and compared to a ground truth expert manual segmentation [25]. The average Dice coefficient of myocardial tissue for all slices in all procedures was 88.0% indicating the clinical validity of the approach. Errors were attributed to low-quality images caused by motion artefacts. Although the segmentation is fully automatic, the clinician has the option to manually edit the result, by clicking on the automatically detected contours in the MR slices, if they are not satisfied with the automatic output. This was unnecessary in 8 of the 14 cases, small changes were made in 5, and larger changes were needed in only 1 case. The average Dice coefficient was 97.0% between the automatic and edited segmentations, indicating that the manual editing is a minor part of the process. The automatic segmentation, along with any possible clinical amendment, takes only a couple of minutes to perform and hence does not disrupt or lengthen the clinical procedure.

**Fig. 2:**
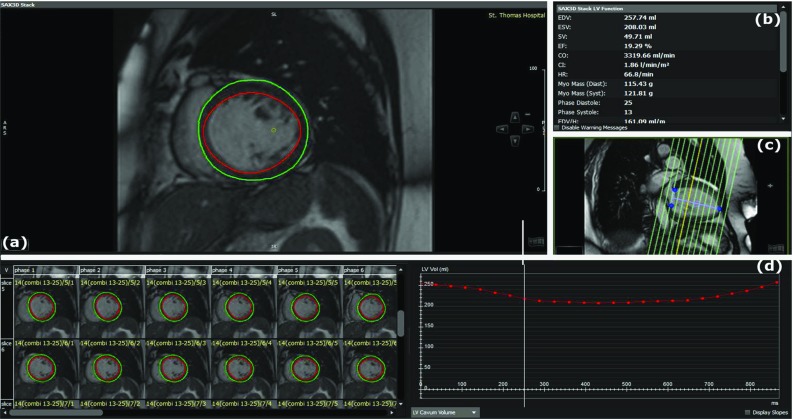
Display of circle CVI42 software, illustrating **a** the slice-by-slice segmentation of the endocardial and epicardial LV borders in the stack of SA, **b** the 3D stack LV function parameters (EDV, ESV, SV and EF), **c** the LV analysis range, and **d** the LV endocardial volume curve over the cardiac cycle

The automatically outlined SA endocardial and epicardial anatomical contours are divided into three layers representing apex, mid and basal. These contours are further divided into segments in each slice following the American Heart Association (AHA) LV model [29]. The area of each of the segments within the slice is then calculated. Based on the segment areas computed and the slice spacing, the volume of each of the 16 segments at each phase of the cardiac cycle is computed in millilitres using Eq. 1.

1 $$\begin{aligned} \hbox {EV} = \sum _\mathrm{slices}\left( \left| \hbox {area}_\mathrm{segment} \right| \times \hbox {slice thickness}\right) \end{aligned}$$

where EV corresponds to the endocardium segment volume. Each of these segment volumes is calculated at each time point, representing the change in volume throughout the cardiac cycle (Fig. 1c). The segment volumes are then added together to compute the global volume throughout the cardiac cycle. LV mechanical dyssynchrony indices based on volumetric analysis are also extracted. These are end-systolic volume (ESV), end-diastolic volume (EDV), stroke volume (SV) and ejection fraction (EF). ESV is the blood volume immediately after contraction, i.e. the minimum volume. EDV is the volume of blood in the ventricle at the end of ventricular diastole. SV is defined as EDV–ESV. EF is the fraction of blood ejected by the LV during systole: $\frac{\mathrm{SV}}{\mathrm{EDV}}*100\%$.

*CMR imaging, offline diagnostic: circle CVI42*

Data were analysed using offline diagnostic software circle CVI42 (version 5.6.4; circle cardiovascular imaging, Canada). Following the pre-procedural CMR acquisition, a fully automatic slice-by-slice segmentation and propagation of the endocardial and epicardial LV borders in the stack of SA and 4-chamber LA SSFP cine images is computed. Manual contour re-positioning is allowed to correct for any misalignment of the segmented SSFP endocardial and epicardial borders (Fig. 2a). Any slices in the stack found beyond the LV are excluded from the LV automatic segmentation (Fig. 2c). Following this, a reference contour for diastole and systole is automatically drawn with the opportunity for manual correction. The software then computes EDV, ESV, SV and EF parameters (Fig. 2b) as well as the global volume curve over the cardiac cycle (Fig. 2d).

*RT3DE, offline diagnostic: TomTec*

**Fig. 3:**
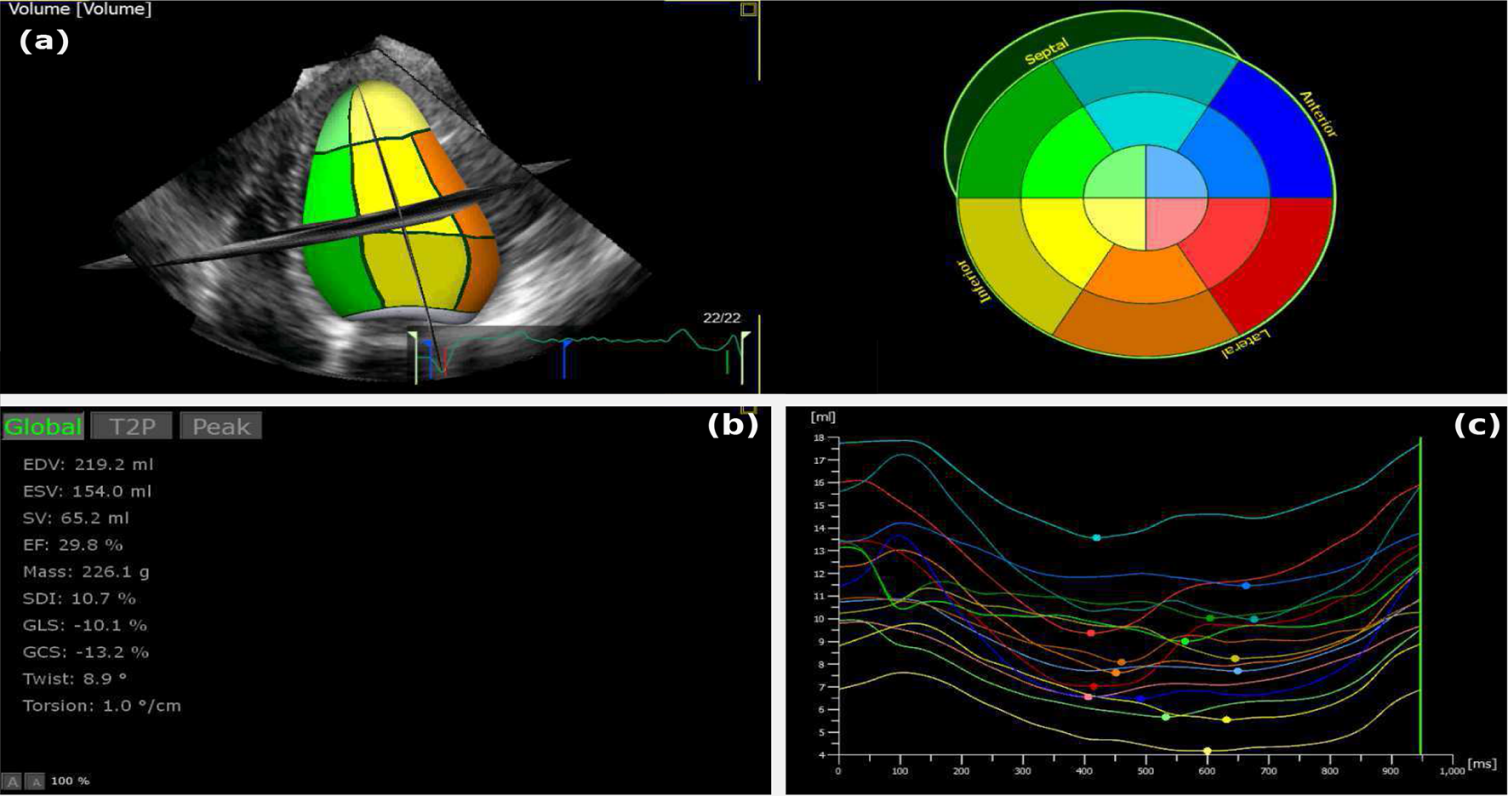
Display of TomTec software illustrating **a** the 3D model of the LV volume, **b** the 3D stack LV function parameters (EDV, ESV, SV and EF) and **c** the regional volume curves

RT3DE acquisitions were analysed with TomTec LV offline diagnostic software (TomTec Imaging Systems GmbH (TOMTEC), Germany). This software performs 3D endocardial border tracking throughout the cardiac cycle, to provide a mathematical model of the LV volume, deriving a time–volume curve. The mitral valve is excluded from the volume calculations. Based on volumetric analysis, EDV, ESV, SV and EF parameters are also extracted (Fig. 3).

**Table 1:** Results of LV function assessment (EDV, ESV, SV and EF) using our interventional system (16 patients/3 volunteers), circle CVI42 (16 patients/3 volunteers) software and TomTec (11 patients) software

	EDV (ml)	ESV (ml)	SV (ml)	EF (%)
Interventional system—16 pat.	$281.10 \pm 68.41$	$229.81 \pm 61.83$	$51.28 \pm 17.86$	$18.58 \pm 6.52$
CVI42—16 pat.	$280.56 \pm 68.84$	$228.54 \pm 62.42$	$52.01 \pm 18.25$	$18.98 \pm 6.77$
TomTec—11 pat.	$238.76 \pm 59.16$	$189.68 \pm 56.48$	$49.08 \pm 16.09$	$21.05 \pm 6.37$
Interventional system—3 vol.	$167.37 \pm 13.44$	$95.63 \pm 9.68$	$71.73 \pm 3.70$	$42.97 \pm 1.30$
CVI42—3 vol.	$167.05 \pm 12.97$	$95.54 \pm 9.59$	$71.51 \pm 3.38$	$42.92 \pm 1.37$

Measurements are presented as means ± SD

### Statistical analysis

*LV function* All data are expressed as mean ± standard deviation. For comparison between EDV, ESV, SV and EF values computed using our interventional system and the other two software systems (circle CVI42 and TomTec), linear regression analysis was performed and a Pearson correlation coefficient (*r*), was calculated. For agreement between our technique and the other two commercially available reference methods, the method of Bland and Altman was used [33] by calculating the mean bias (mean difference) and the 95% limits of agreements (variability was expressed as mean difference ± 2 SD between the two measurements). The mean coefficient of variation (CoV), defined as standard deviation (SD)/Mean, was also calculated. A Pearson correlation coefficient of $>0.80$ or a CoV $<10\%$ was considered as excellent agreement. A value of $p <0.05$ was considered statistically significant.

**Table 2:** Pearson correlation coefficients (*r*), *p* values and coefficient of variation for EDV, ESV, SV and EF between our interventional system and the two commercially available techniques

	Interventional system versus CVI42				Interventional system versus TomTec			
	EDV (ml)	ESV (ml)	SV (ml)	EF (%)	EDV (ml)	ESV (ml)	SV (ml)	EF (%)
Pearson Corr.	0.980	0.984	0.996	0.993	0.723	0.755	0.639	0.665
*p* values	$1.013^{-12}$	$1.950^{-13}$	$7.339^{-18}$	$1.6184^{-16}$	0.0120	0.0073	0.0343	0.0256
CoV (%)	0.173	0.310	0.896	0.943	8.277	11.038	11.066	11.091

In total 16 patients and 3 volunteers were processed for the comparison between our interventional system and circle CVI42 and 11 patients were processed for the comparison between our interventional system and TomTec software

**Table 3:** Mean bias (mean difference) and the 95% limits of agreement (variability is expressed as mean difference ± 1.96 SD between the two measurements) for EDV, ESV, SV and EF after comparing our interventional system to the other two software systems, circle CVI42 (16 patients and 3 volunteers) and TomTec (11 patients)

	Interventional system versus CVI42				Interventional system versus TomTec			
	EDV (ml)	ESV (ml)	SV (ml)	EF (%)	EDV (ml)	ESV (ml)	SV (ml)	EF (%)
Mean bias	0.530	1.086	$-$ 0.565	$-$ 0.321	28.195	28.032	0.146	$-$ 2.214
$+\,1.96$ SD	3.029	3.684	1.619	0.468	108.375	100.664	28.868	9.333
$-\,1.96$ SD	$-$ 1.969	$-$ 1.511	$-$ 2.749	$-$ 1.109	$-$ 51.984	$-$ 44.600	$-$ 28.575	$-$ 13.760

*LV global volume* For each of the three techniques, the global volume curves over the cardiac cycle were plotted and compared using correlation coefficients (CC). Additionally, for all three techniques, the time to maximal contraction is calculated and is expressed as a percentage of the total cardiac cycle.

## Results

*LV function* Table 1 illustrates the mean ± SD of LV volumes (ESV, EDV, SV) and EF for all patients and volunteers computed using all three techniques. Comparison of our approach to each of the two commercially available reference approaches is found in Tables 2 and 3. Table 2 demonstrates the Pearson correlation coefficients, *p* value and CoV, while Table 3 demonstrates Bland–Altman results (mean bias and 95% limits of agreement). As indicated in Table 2, an excellent statistically significant correlation was found between our interventional system and circle CVI42 ($r >0.980$, $p <1.02^{-12}$) for all four parameters. Regarding the comparison between our interventional system and TomTec, a good statistically significant correlation was found for all parameters ($0.6< r< 0.8$, $p <0.05$). In addition, CoV was found to be < 1% when compared to circle CVI42 and $8\%< \hbox {CC}<12\%$ when compared to TomTec, indicating an excellent and a good agreement, respectively.

**Fig. 4:**
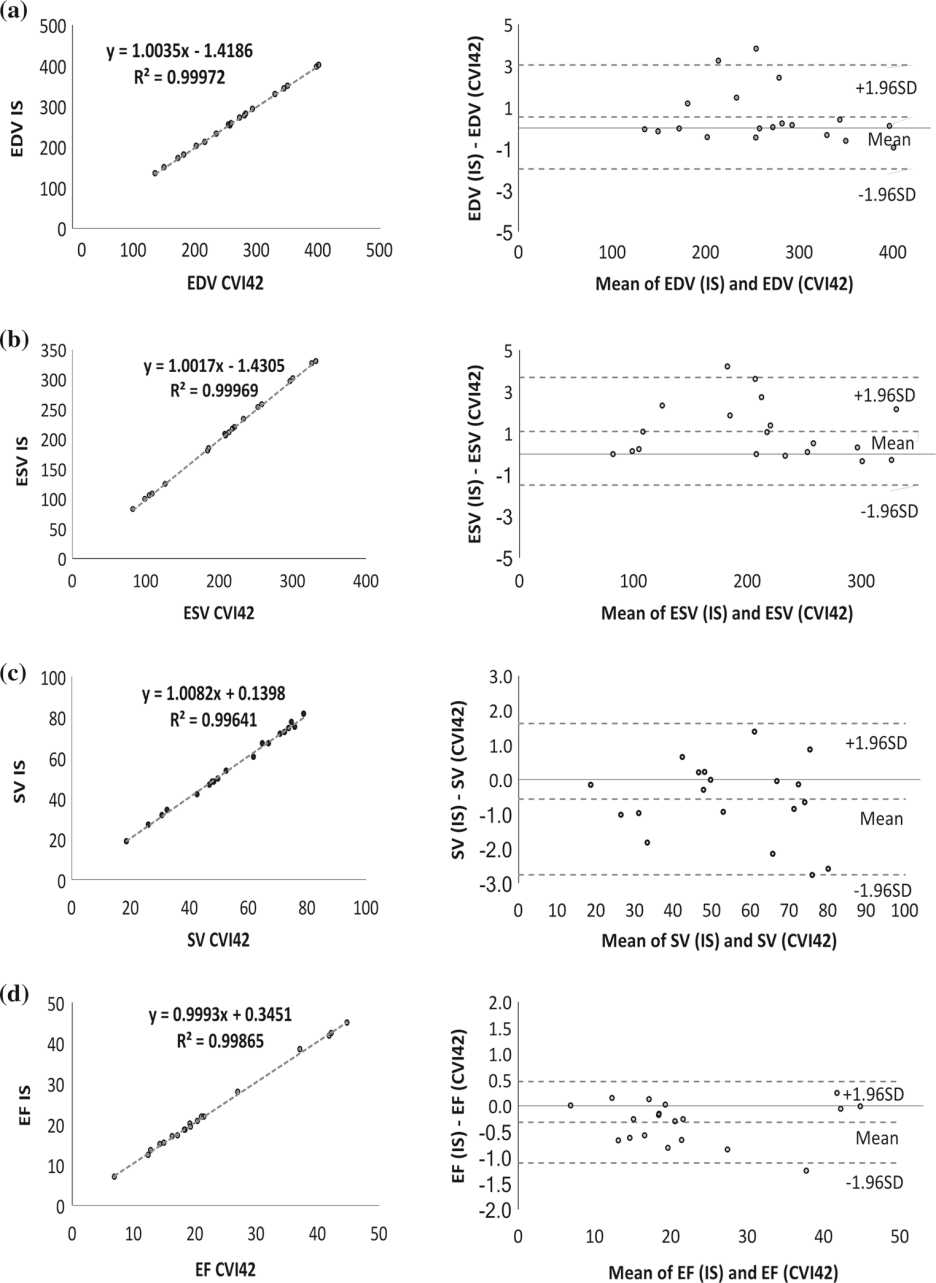
Linear regression (left) and Bland–Altman (right) diagrams of comparison between our interventional system (IS) and circle CVI42 for the assessment of **a** EDV, **b** ESV, **c** SV and **d** EF parameters

**Fig. 5:**
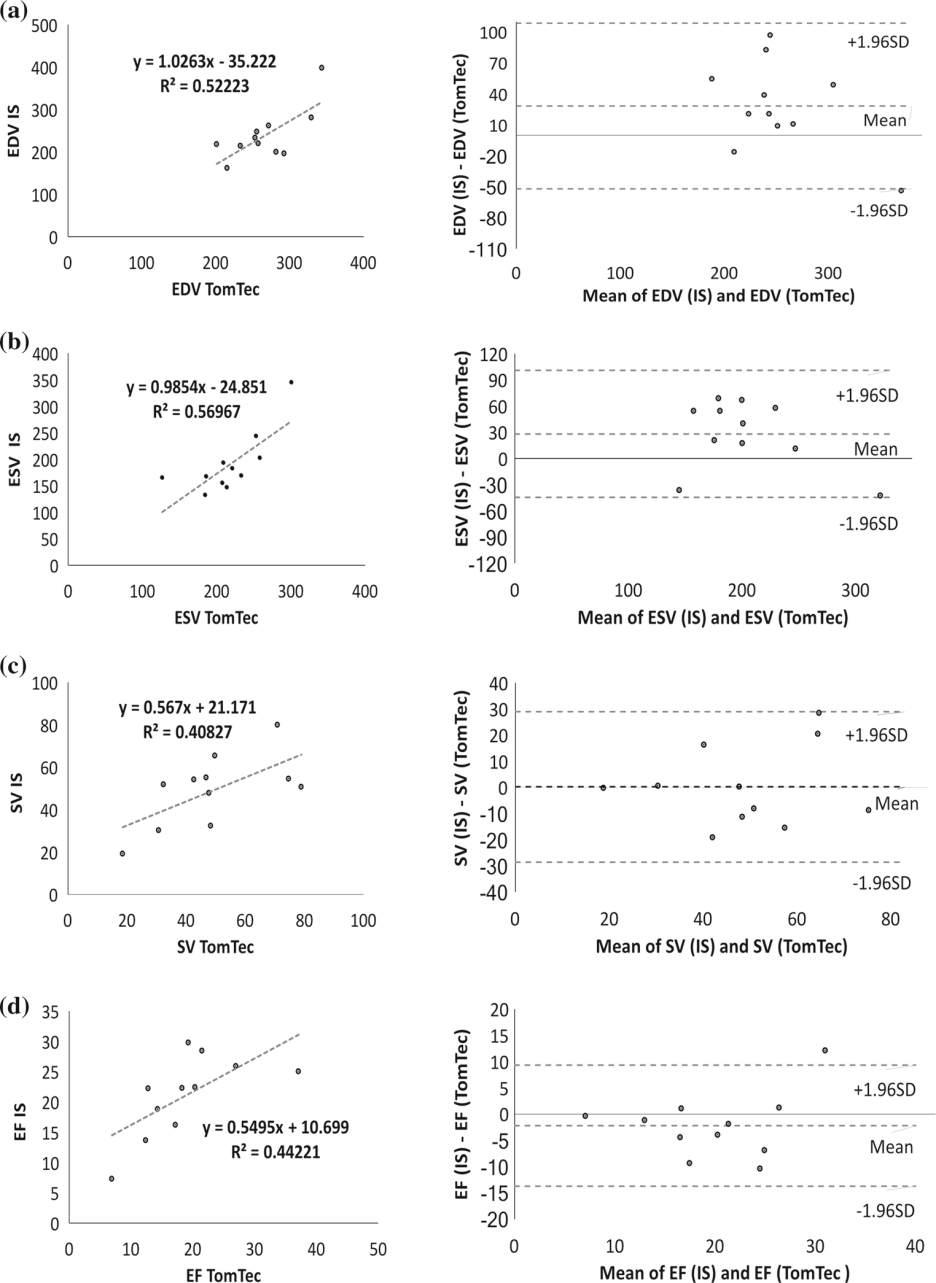
Linear regression (left) and Bland–Altman (right) diagrams of comparison between our interventional system (IS) and TomTec for the assessment of **a** EDV, **b** ESV, **c** SV and **d** EF parameters

Figure 4 illustrates the results of linear regression analysis (left) and Bland–Altman (right) for EDV, ESV, SV and EF between our interventional system and circle CVI42, while Fig. 5 illustrates linear regression analysis (left) and Bland–Altman (right) for EDV, ESV, SV and EF between our interventional system and TomTec. Circle CVI42 dyssynchrony indices correlate well with the values from our interventional system, obtaining $R^{2}$ values of $>0.99$ for ESV, EDV, SV and EF parameters. Linear models between TomTec parameters and our interventional system showed lower $R^{2}$ values of $0.4< R^{2}<0.6$. Bland–Altman analysis showed significantly high agreement between our interventional system and circle CVI42, revealing significantly narrow limits of agreement for all parameters and only minimal mean bias, indicating concordance between both methods. Bland–Altman plots demonstrated relatively wider limits of agreement with higher mean bias for all parameters when compared to the TomTec software. Overall, RT3DE underestimated end-systolic and end-diastolic volumes, resulting in a slight overestimation of ejection fractions when compared with CMR.

**Fig. 6:**
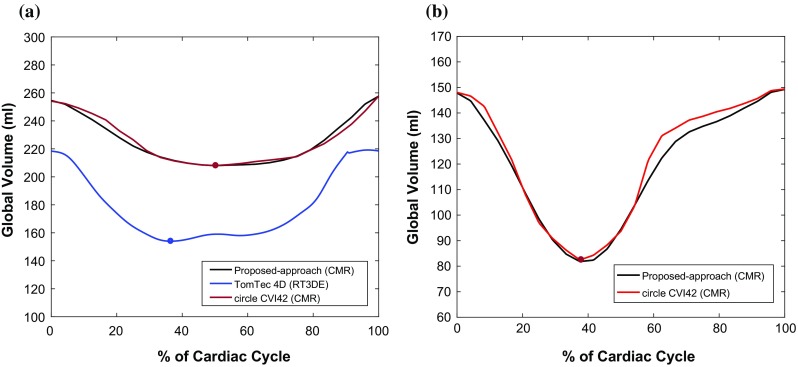
Global volume curves over the cardiac cycle for **a** one example patient and **b** one volunteer. The time to maximal contraction is also illustrated on the graphs, with a circle

*LV global volume* Figure 6 illustrates the global volume curves over the cardiac cycle for (a) one example patient and (b) one volunteer for each of the techniques used. Table 4 illustrates the median correlation coefficient and interquartile range (*Q*3–*Q*1) of the global volumes for all patients/volunteers between our interventional system and the two alternative techniques. Regarding the % of cardiac cycle to minimum volume, for all patients/volunteers, found using our interventional system and the circle CVI42 software, we found a complete agreement; that is, the minimum volumes of all patients/volunteers occurred at the same point of the cardiac cycle using both techniques. When compared to TomTec, the average difference was computed to be 5.750 ± 3.690 (%).

## Discussion and conclusion

Computer-assisted interventional systems have greater demands compared to offline diagnostic software. The interventional systems must be fast, highly automated and easy to use for interventional clinicians who may not be experts in interpreting pre-operative imaging. In this study, the accuracy of an interventional system [3, 25] is validated against offline diagnostic systems. LV function metrics are quantitatively evaluated against two commercially available diagnostic software systems, circle CVI42 (CMR) and TomTec (RT3DE).

Our results confirm a strong agreement between CMR evaluation of EDV, ESV, SV and EF parameters. Specifically, a Pearson correlation coefficient of $> 0.98$ (*p* value $<\,1.02^{-12}$) was found for all parameters as well as a CoV of < 1%. Linear regression analysis indicated that our interventional system correlates well with the other CMR technique, circle CVI42, obtaining $R^{2}$ values of $>0.99$ for all parameters. Finally, Bland–Altman analysis demonstrated concordance between both CMR methods, with tight limits of agreement and only minimal mean biases, again for all parameters. In relation to global left ventricular volumes over the cardiac cycle, an excellent median correlation coefficient of 0.983 (interquartile range = 0.030) was found, while a perfect agreement was found for % of cardiac cycle to minimum volume for all patients/volunteers.

On the contrary, a less than linear relationship was found between our interventional system and RT3DE, as reflected by the lower Pearson correlation coefficients ($0.6< r <0.8$, $p <0.05$) and the higher CoV ($8\%< CoV<12\%$), clinically interpreted as good agreement. Linear regression analysis demonstrated a moderate correlation between our interventional system and RT3DE, showing $R^{2}$ index values of $0.4< R^{2}<0.6$ for the four parameters. CMR overestimated end-systolic and end-diastolic volumes, resulting in a slight underestimation of ejection fractions when compared with RT3DE approach as found by Bland–Altman analysis. This is probably due to the way TomTec software determines LV volumes. The determination of LV volumes is based on the geometric centre-point, i.e. the LV is subdivided into pyramidal volumes with the base corresponding to one of the 16 segments and the apex the centre-point. This inevitably means the sum of subvolumes is less than the actual volume. Even though this difference in the computation of LV volumes exists between the two techniques global left ventricular volumes over the cardiac cycle showed an excellent median correlation coefficient of 0.944 (interquartile range = 0.095) in relation to our interventional system, while a 5.750 ± 3.690 average difference was found for % of cardiac cycle to minimum volume for all patients.

**Table 4:** Correlation coefficients between our interventional system and the two software systems, circle CVI42, comparing 16 patients and 3 volunteers and TomTec, comparing 11 patients

	Median correlation coeff.	Interquartile range (*Q*3–*Q*1)
Interventional system versus CVI42	0.983	0.030
Interventional system versus TomTec	0.944	0.095

A limitation of the study was the relatively small size of our study group. Future work will focus on increasing considerably the number of patients and volunteers included as well as using patients that undergo both RT3DE and CMR imaging within 1 day to minimise changes in cardiac function. Additionally, we are planning to compute a regional (per segment) volume evaluation as well as validation with a manual ground truth annotation over all cardiac cycles. We are also beginning a multi-centre study of the system to more thoroughly evaluate its clinical value.
